# Role, race, and place: Prostate cancer disparities in Patients' and Partners' health outcomes and psychosocial factors

**DOI:** 10.1002/cam4.5646

**Published:** 2023-02-07

**Authors:** Lixin Song, Thomas C. Keyserling, Ronald C. Chen, Chunxuan Ma, Shenmeng Xu, Karl Shieh, Gail P. Fuller, Matthew E. Nielsen, Laurel L. Northouse, Xianming Tan, Christine Rini

**Affiliations:** ^1^ School of Nursing University of Texas Health Science Center at San Antonio (UTHSCSA) San Antonio Texas USA; ^2^ Mays Cancer Center UTHSCSA San Antonio Texas USA; ^3^ School of Medicine University of North Carolina‐Chapel Hill (UNC‐CH) Chapel Hill North Carolina USA; ^4^ University of Kansas Medical Center Kansas City Kansas USA; ^5^ Jean and Alexander Heard Libraries, Digital Scholarship and Communications Vanderbilt University Tennessee Nashville USA; ^6^ School of Nursing UNC‐CH Chapel Hill North Carolina USA; ^7^ Lineberger Comprehensive Cancer Center UNC‐CH Chapel Hill North Carolina USA; ^8^ School of Nursing University of Michigan Ann Arbor Michigan USA; ^9^ Gillings School of Global Public Health UNC‐CH Chapel Hill North Carolina USA; ^10^ Cancer Survivorship Institute and Department of Medical Social Sciences Northwestern University Evanston Illinois USA

**Keywords:** area deprivation index (ADI), health disparities, prostate cancer, psychosocial factors, quality of life, stress coping, symptom

## Abstract

**Purpose:**

This study aimed to examine the effects of participant role (patient vs. partner), race (white vs. non‐white), and place (less vs. more neighborhood deprivation) on health outcomes (quality of life [QOL] and symptoms) and stress‐coping‐related psychosocial factors (appraisals of illness and coping resources).

**Methods:**

This descriptive study included 273 patients and their partners (dyads) who transitioned from PCa treatment to self‐management. We used established, psychometrically sound measures to assess health outcomes and psychosocial factors and conducted multilevel modeling analyses.

**Results:**

Compared to partners, patients reported worse physical QOL; less frequent anxiety; less pain and fatigue; less bothersome hormonal problems; more bothersome urinary and sexual problems; greater self‐efficacy; and more instrumental support. Compared to their white counterparts, non‐white dyads reported better overall, emotional, and functional QOL; less depression; more positive appraisals, and greater self‐efficacy. Compared to dyads in low ADI neighborhoods, dyads in high ADI (more deprived) neighborhoods reported worse social QOL; more bothersome urinary, sexual, and hormonal symptoms; and less interpersonal support. White patients reported the highest emotional support among all groups, while white partners reported the lowest emotional support.

**Conclusion:**

Our findings underscore the need to consider social determinants of health at multiple levels when investigating PCa disparities. Considering neighborhood‐level socioeconomic factors, in addition to race and role, improves our understanding of the PCa disparities in QOL, symptoms, and psychosocial factors among patients and partners. Targeted multilevel supportive care interventions should tailor to the needs of racially diverse PCa patients and partners residing in deprived neighborhoods are needed.

## Graphical abstract

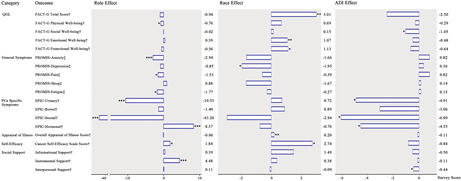

## 1 INTRODUCTION

Disparities in prostate cancer (PCa hereafter) are well described across different patient groups.^1^ Factors previously examined to explain the disparities include the disparities related to race (Black vs. non‐Hispanic white) and socioeconomic status in PCa incidence and mortality rates,^1^, ^2^, ^3^, ^4^, ^5^, ^6^, ^7^, ^8^, ^9^, ^10^ PCa risks, stage and aggressiveness, and survival.^11^, ^12^ However, little research has examined PCa disparities in survivorship experiences (quality of life (QOL), symptoms, and psychosocial factors) among patients of different racial backgrounds and their partners, especially how multiple social determinants of health synergistically influence the disparities. Understanding PCa disparities in survivorship experiences among patients and their partners are important because PCa is considered a “couple's illness.” As the primary caregiver for PCa patients in an intimate relationship, partners play an integral role in providing care and support.^13^ Adverse effects of PCa and treatment‐related symptoms on partner QOL and psychosocial factors may be as great or even greater than the effects on patient QOL.^14^, ^15^

This study has expanded the abovementioned prior investigations and examined PCa disparities in health outcomes (e.g., QOL and symptoms) and psychosocial factors in the context of *socioeconomic deprivation* among patients with different racial backgrounds and their partners. Socioeconomic deprivation is a complex theoretical construct that allows for a sophisticated assessment of the impact of exposure to multiple social determinants of health. It captures a person's state of being challenged at the individual and community levels by incorporating a combination of factors, including low income, limited education, substandard living conditions, and neighborhood factors.^16^ Although managing PCa and related issues involve the social, economic, and physical environment at the individual, family, and neighborhood levels, little research has examined whether simultaneous exposure to the adverse conditions associated with living in a deprived neighborhood influence the survivorship experiences of patients and partners.^16^

In this study, we assessed socioeconomic deprivation at the neighborhood level using the Area Deprivation Index (ADI). The ADI is a multidimensional measure as a proxy for social, economic, and physical conditions in census blocks using 9‐digit zip codes—the smallest geographical unit used by the US Census Bureau. We examined the effects of role (patient vs. partner), race (white vs. non‐white), and place (ADI, more vs. less neighborhood deprivation) on the health outcomes (QOL and symptoms) and psychosocial factors (appraisals of illness and coping resources) of localized PCa patients and their partners.

## 2 METHODS

This study used the baseline data of a randomized controlled trial (RCT) (Clinicaltrial.gov ID: NCT03489057). The RCT aimed to test the efficacy of a tailored eHealth symptom management intervention on the QOL and the stress‐coping processes of patients with localized PCa and their intimate partners.^17^

### 2.1 Study sample

Patients were eligible if: they were 40–75 years of age, within 16 weeks of completing treatment with curative intent for localized PCa (confirmed by hospital medical records), had no prior cancer within the past 2 years, had no concurrent cancer (excluding non‐melanomatous skin cancer). Patients also had to have an intimate partner (male or female) who was 18 years or older, willing to participate, and not diagnosed with cancer or receiving treatment for cancer within the past 12 months. Patients and partners had to read and speak English and had no significant cognitive impairments.

### 2.2 Recruitment

The recruitment process has been detailed previously.^17^ After the Institutional Review Board approval, we recruited patient‐partner dyads using the North Carolina Central Cancer Registry (NCCCR) Rapid Case Ascertainment, state‐wide collection of new prostate cancer cases in each county within a week of diagnosis. Our recruitment method allowed us to obtain a population‐based sample and expand our geographic catchment area to the state population of prostate cancer patients, especially targeting the 36 counties with the highest proportions of ethnic populations and poverty. We mailed introduction brochures about the study, informed consent information, and opt‐out letters to patients' physicians and then to patients subsequently, giving each three and two weeks, respectively, to request that a patient not be approached for study inclusion. After this opt‐out period, we called to assess patients' interest in participating, answered questions, and screened for eligibility. After eligible patients permitted us to contact their partners, we called the partners to screen for their eligibility. Based on participants' preferences, we obtained informed consent from patients and partners separately via telephone, mail, or DocuSign.

Over 3 years (from 2018 to 2021), we obtained the names of 3078 patients from NCCCR. We screened 2899 patients for their eligibility, contacted 2195 eligible patients to obtain permission to approach their intimate partners, and successfully approached 357 partners. Two hundred eighty patients and their partners (i.e., dyads/couples) met our inclusion criteria, consented to the study, and completed the baseline assessments (enrollment rate, 12.78%). The current study analyzed the baseline data of 273 dyads who provided valid 9‐digit zip codes for ADI calculation.

### 2.3 Measurement

Patients and partners separately completed the following assessments via telephone or online survey based on their preferences.

*Health Outcomes* included cancer‐related QOL and symptoms. The 27‐item Functional Assessment of Chronic Illness Therapy General Scale (FACT‐G) assessed c*ancer‐related QOL* and the *physical, social, emotional, and functional subdomains of QOL*.^18^, ^19^ Partners reported their own QOL using the partner's version of FACT‐G.^20^

PROMIS measures of cancer anxiety,^21^ depression,^22^ pain,^23^, ^24^ sleep disturbance,^25^ and fatigue^26^ assessed *general symptoms*. Patients and partners separately reported the severity of their own pain and sleep disturbance and the frequency of anxiety, depression, and fatigue. We measured *prostate cancer‐specific symptoms* with the Prostate Cancer Index Composite (EPIC‐26),^27^ which evaluates the patients' function and symptom‐specific bother, including urinary, bowel, sexual, and hormonal symptoms. Partners completed a four‐item EPIC‐Spouse^14^ to assess how much of a bother/burden the patients' symptoms were for the partners.

*Psychosocial factors* included appraisals of illness and coping resources (self‐efficacy and social support). *Appraisals of illness*—patients' and partners' perceptions of the degree of threat associated with PCa—were assessed using an adapted 20‐item, 5‐point Likert response Appraisal Scale.^28^, ^29^ *Self‐efficacy in symptom management*—confidence in managing PCa—was assessed using a 9‐item modified version of the Lewis Cancer Self‐Efficacy Scale (CASE).^30^ PROMIS Emotional,^31^ Informational,^32^ and Instrumental Support Measures,^33^ and the Interpersonal Support Subscale^34^ assessed s*ocial support* from people other than the partner.

### 2.4 Other factors

We obtained demographic data, including race, ethnicity, and home zip code, from all participants and cancer‐related information from patient medical charts and self‐reports.

### 2.5 Data analysis

We used descriptive statistics to describe the demographics, medical characteristics, and study variables of the PCa patients and their partners. The Area Deprivation Index (ADI) is a validated index that ranks a neighborhood's socioeconomic deprivation^35^ using 17 measures, including education, employment, housing quality, and poverty.^36^ We linked the 9‐digit zip codes of patients' home addresses to ADI rankings for their census block group using the United States Postal Service Web Tools Application Programming Interface Portal and the Neighborhood Atlas® ‐ 2019 ADI (https://www.neighborhoodatlas.medicine.wisc.edu/). We used the same ADI for each dyad because 98% of the patients lived in the same household as their partners. The ADI value ranged from 1 to 10, a higher score indicating a more disadvantaged neighborhood than other block groups within North Carolina. Based on the median ADI of our study sample, we dichotomized the ADIs into “low” (ADI <= 3, less deprived) or “high” (ADI > 3, more deprived).^37^ We used chi‐square and t‐tests to separately compare demographic differences between low ADI vs. high ADI for patients and partners.

**FIGURE 1:**
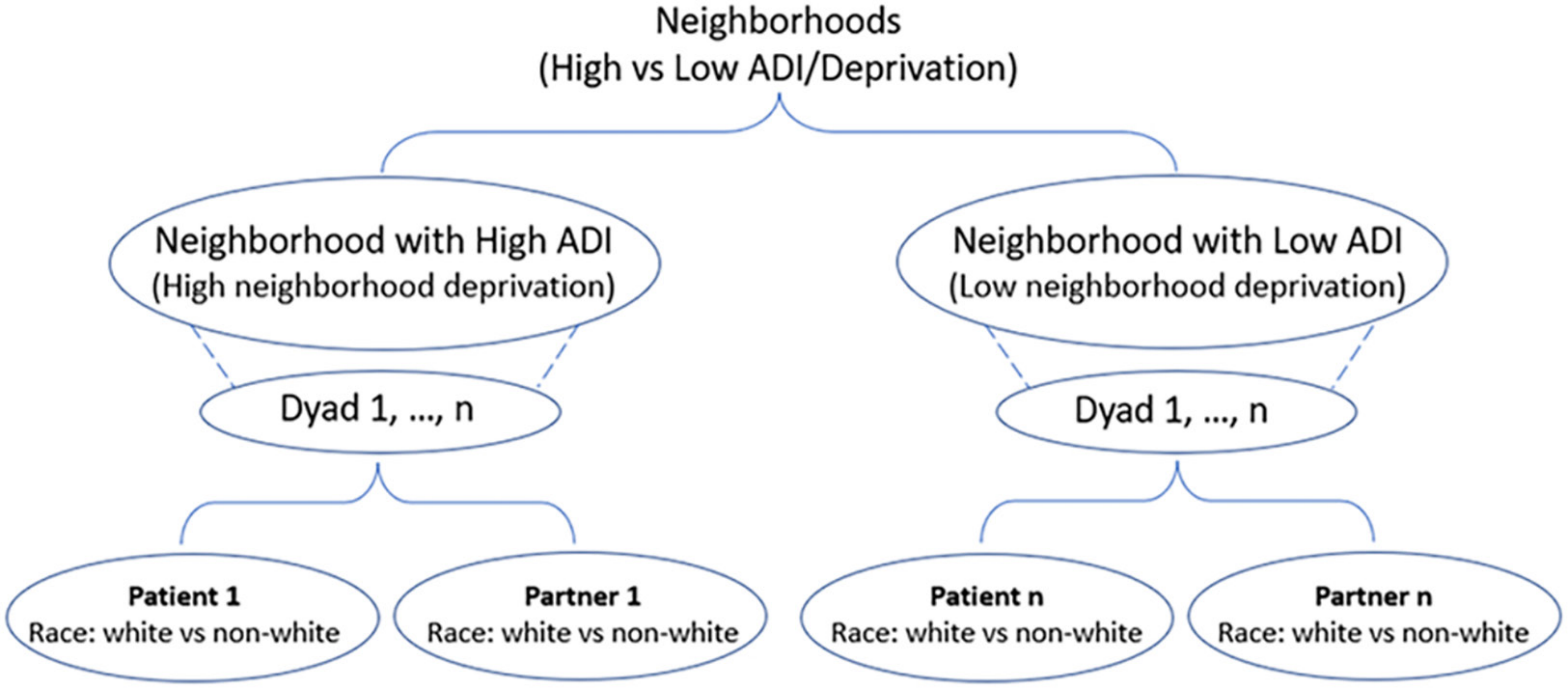
Data structure hierarchy.

We performed multilevel modeling (MLM) analyses with cluster data^38^ to examine the effects of role, race, and ADI on health outcomes and psychosocial factors (Figure 1 shows the data hierarchy). We used MLM to achieve the research aims because the outcome variables of patients and partners in the same dyads are correlated as they share some common dyad‐specific features (e.g., ADI). MLM allows for estimating the fixed effects of dyad‐level predictor (ADI) and the individual‐level predictors (race, role) and the unobserved random effects of dyad characteristics simultaneously.^39^ Interpretation of the fixed effects is the same as interpreting regression coefficients in linear regression. To fit a final MLM model, the initial models fitted for each variable also included the main effects of role, race, and ADI, and their 2‐way and 3‐way interactions. If nonsignificant, we removed the 3‐way interaction (role*race*ADI) and examined the 2‐way interactions. If nonsignificant, we removed the 2‐way interactions and examined the main effects of role, race, and ADI in the final models. To control for the effects of potential covariates, we also included in the initial model all factors relevant to the health outcomes and psychosocial factors collected in this study. These factors included ethnicity, education, family income, number of people supported by the income, employee status, age, CCI, patient–partner relationship, prostate cancer‐specific antigen (PSA), and Gleason score. After removing all nonsignificant covariates, the following factors were significant and included in the final MLMs, that is, age, education, ethnicity, employment status (presently working), comorbid conditions (CCI), the type of cancer treatment, and Gleason score. Based on the final parsimonious models, we calculated the least‐square means and compared their differences in study variables among the four groups (white vs. non‐white patients and white vs. non‐white partners). All tests were 2‐sided at alpha level 0.05 using SAS (version 9.4, SAS Institute, Inc., Cary, NC).

## 3 RESULTS

### 3.1 Participant characteristics

Three‐quarters of patients had a prostatectomy. Approximately 77.66% of the patients and 77.12% of the partners self‐reported their race as white. Among the 118 and 155 patient–partner dyads who lived in high vs. low ADI areas, respectively, whites were more likely to live in less deprived neighborhoods, and non‐whites were more likely to live in more deprived neighborhoods (*p* = 0.03). Compared to those in less deprived level neighborhoods, patients and partners in more deprived areas were more likely to report lower education (*p* < 0.001 and *p* = 0.03, respectively), and a family income of less than $90,000 (*p* < 0.001). Although no significant differences were detected in the PSA and Gleason scores among patients in low and high ADI areas (*p* = 0.07 and 0.38, respectively), patients in less deprived neighborhoods were more likely to have had a prostatectomy. In contrast, those in more deprived neighborhoods were more likely to have received radiation therapy (*p* = 0.02). Patients and partners in high ADI areas reported significantly more comorbidities than those in low ADI (*p* = 0.01 and 0.04, respectively) (Table 1).

**TABLE 1:** Participant demographics.

Characteristics	Patients							Partners						
	Overall (*N* = 273)		High ADI^a^ (More deprived neighborhood) (*N* = 118)		Low ADI (Less deprived neighborhood) (*N* = 155)		*p*‐value^f^	Overall (*N* = 273)		High ADI (More deprived neighborhood) (*N* = 118)		Low ADI (Less deprived neighborhood) (*N* = 155)		*p*‐value^f^
	*N*	%^d^, ^e^	*N*	%^d^, ^e^	*N*	%^d^, ^e^		*N*	%^d^, ^e^	*N*	%^d^, ^e^	*N*	%^d^, ^e^	
**Gender**														
Male	273	100.00	118	43.22	155	56.78	NA	1	0.37	0	0.00	1	100.00	1.00
Female	0	0.00	0	0.00	0	0.00		272	99.63	118	43.48	154	56.62	
**Race** ^c^														
White	212	77.66	84	39.62	128	60.38	0.03	209	77.12	87	41.63	122	58.37	0.35
Non‐white	61	22.34	34	55.74	27	44.26		62	22.88	30	48.39	32	51.61	
**Ethnicity** ^c^														
No	264	97.78	114	43.18	150	56.82	1.00	262	97.04	116	44.27	146	55.73	0.47
Yes	6	2.22	3	50.00	3	50.00		8	2.96	2	25.00	6	75.00	
**Education** ^c^														
Less than college	108	39.71	70	64.81	38	35.19	<0.001	108	39.56	53	49.07	55	50.93	0.03
Bachelor's Degree	82	30.15	14	17.07	68	82.93		81	29.67	28	34.57	53	65.43	
Graduate Degree^b^	66	24.26	23	34.85	43	65.15		60	21.98	22	36.67	38	63.33	
Other	16	5.88	11	68.75	5	31.25		24	8.79	15	62.50	9	37.50	
**Family income** ^c^														
<=$90,000	127	47.04	74	58.27	53	41.73	<0.001	119	44.07	66	55.46	53	44.54	<0.001
>$90,000	130	48.15	38	29.23	92	70.77		128	47.41	40	31.25	88	68.75	
Do not know/refused	13	4.81	5	38.46	8	61.54		23	8.52	10	43.48	13	56.52	
**Presently working** ^c^														
Yes	140	51.47	55	39.29	85	60.71	0.08	135	49.45	58	42.96	77	57.03	0.62
**Type of treatment**														
Surgery	202	73.99	79	39.11	123	60.89	0.02	–	–	–	–	–	–	–
Radiation	71	26.01	39	54.93	32	45.07								
**Gleason scores**														
Grade Group 1	39	14.34	17	43.59	22	56.41	0.38	–	–	–	–	–	–	–
Grade Group 2	108	39.71	39	36.11	69	63.89								
Grade Group 3	71	26.10	36	50.70	35	49.30								
Grade Group 4	34	12.5	15	44.12	19	55.88								
Grade Group 5	20	7.35	10	50.00	10	50.00								

	Mean	SD	Mean	SD	Mean	SD	*p*‐value	Mean	SD	Mean	SD	Mean	SD	*p*‐value
Age	63.85	6.65	63.86	7.00	63.85	6.39	0.99	61.17	7.38	60.71	7.01	61.52	7.65	0.37
PSA	2.39	1.46	2.52	1.44	2.30	1.46	0.22	–	–	–	–	–	–	–
Charlson Comorbidity Index	4.23	1.90	4.55	1.99	3.98	1.79	0.01	3.79	2.00	4.08	1.99	3.57	1.99	0.04
Patient‐partner relationship (Year)	33.11	12.67	32.23	12.49	33.79	12.81	0.32	33.49	12.61	32.76	12.06	34.05	13.03	0.41
Number of people supported by income	2.43	0.93	2.46	1.05	2.41	0.82	0.67	2.49	1.02	2.51	1.16	2.46	0.90	0.64

^a^ ADI (area deprivation index) was categorized into two groups, high ADI (ADI > 3) and low ADI (ADI <= 3).

^b^ Graduate Degree: include both master's and doctoral‐level degrees.

^c^ The total number at each level was non‐identical to the overall number because some participants did not respond to questions.

^d^ Percentages have been rounded and may not total 100.

^e^ The overall demographics by role are shown as column percentage, the participants' demographics by ADI and role are shown as row percentage.

^f^ *p*‐value of categorical variables obtained from chi‐square test. The *p*‐values of continuous variables were obtained from t‐test. A *p*‐value 0.05 is considered significant.

### 3.2 Cancer‐related QOL

**FIGURE 2:**
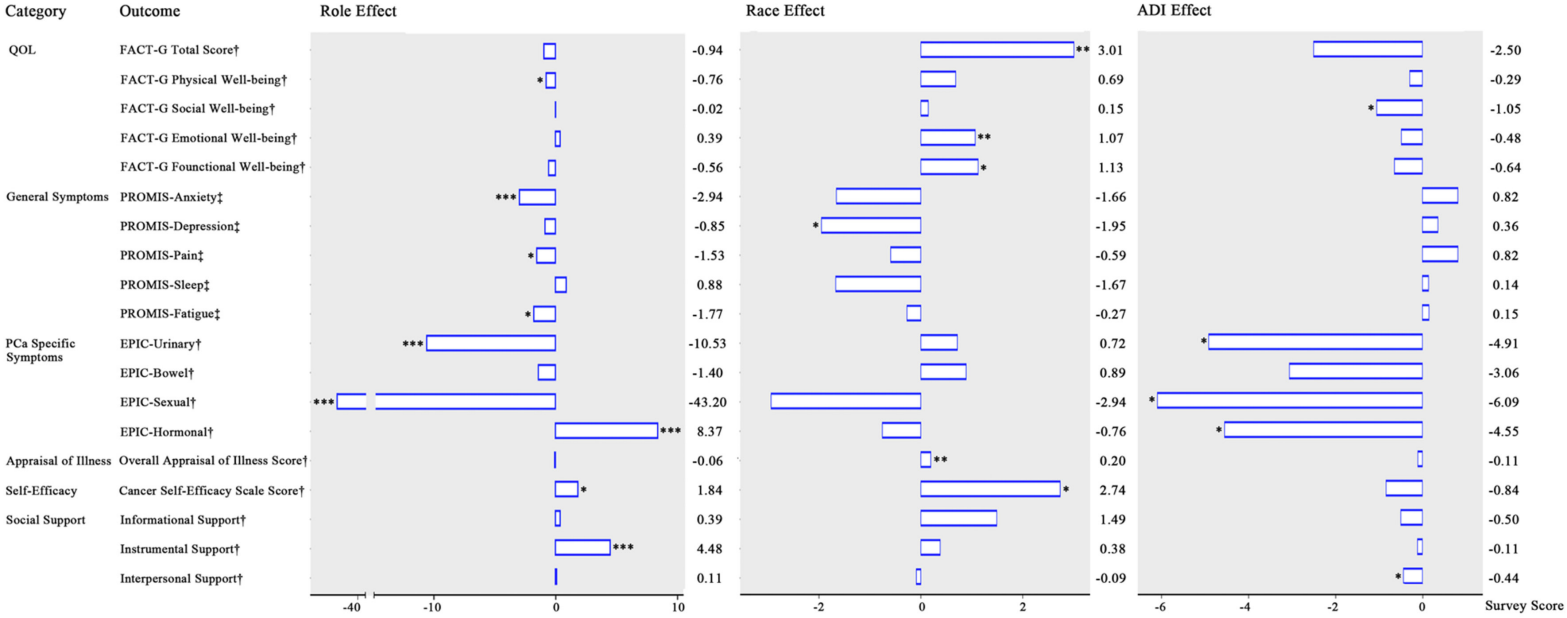
Effects of role, race, and ADI on prostate cancer disparities. Model results of the main effects after removing the nonsignificant 2‐way and 3‐way interactions. Referents—role effect: partner; race effect: white; ADI effect: low ADI. Estimate <0 means the referents' (partner, low ADI, and white participants) scores were greater than their comparators. ADI (area deprivation index) was categorized into two groups, high ADI (ADI > 3, more deprived neighborhoods) and low ADI (ADI <= 3, less deprived). ^†^Higher scores indicated more positive results; ^‡^Higher scores indicated more negative results. EPIC scores for partners (ranging from 1 to 5) were standardized using the same approach as recommended by EPIC‐26 to be consistent with patients' scores (range, 0–100). *p < 0.05, **p < 0.01, ***p < 0.001. QOL, quality of life; FACTG, The Functional Assessment of Cancer Therapy ‐ General; PROMIS, The Patient‐Reported Outcomes Measurement Information System; EPIC, Expanded Prostate Cancer Index Composite short form.

The only significant role effect was on physical well‐being. Compared to partners, patients reported worse physical QOL (*p* < 0.05) (Figure 2 and Appendix S1). There are significant race main effects on total and emotional, and functional subdomains of QOL. Compared with whites, non‐white participants reported better overall QOL as well as emotional (both *p*s < 0.01) and functional QOL (*p* < 0.05). Analyses revealed significant ADI main effects on social well‐being. Compared with participants in low ADI neighborhoods, dyads in high ADI neighborhoods had worse social well‐being (*p* < 0.05). (Note: All MLM results are shown in Appendix S1 unless indicated otherwise).

### 3.3 Symptoms

Results showed significant role, race, and ADI main effects on both general and PCa‐specific symptoms. Compared to partners, patients reported less frequent anxiety (*p* < 0.001), less pain (*p* < 0.05) and fatigue (*p* < 0.05); fewer bothersome PCa‐related hormonal problems (*p* < 0.001); but more bothersome urinary and sexual symptoms (both *p*s < 0.001). Compared to their white counterparts, non‐white participants reported less depression (*p* < 0.05). Compared to dyads in less deprived neighborhoods, those in more deprived areas reported more bothersome patients' urinary, sexual, and hormonal symptoms (all *p*s < 0.05). Taking the anxiety symptom as an interpretation example: The MLM analysis showed significant role effect (*p* < 0.001), that is, patients' anxiety mean score was 2.94 points lower as compared to their partners', indicating the partners more frequently reported anxiety. However, the MLM analysis did not find significant race (*p* = 0.08) and ADI effects (*p* = 0.35), indicating that the anxiety scores were similar among white vs. non‐white dyads and dyads in the high vs. low ADI neighborhoods (Figure 2).

### 3.4 Appraisals of illness

Results showed significant race main effects on Appraisals of Illness. Non‐white patients and partners reported more positive appraisals compared to their white counterparts (*p* < 0.01).

### 3.5 Self‐efficacy

Results showed significant role and race main effects on self‐efficacy. Patients reported greater confidence in managing PCa as compared to their partners (*p* < 0.05). Non‐white patients and partners reported greater self‐efficacy compared to their white counterparts (*p* < 0.05).

### 3.6 Social support

Results showed significant role effects on instrumental support and ADI effects on interpersonal support. Patients reported higher instrumental support (*p* < 0.001) compared to partners. Compared to dyads in low ADI neighborhoods, those in high ADI neighborhoods reported less interpersonal social support (*p* < 0.05).

**FIGURE 3:**
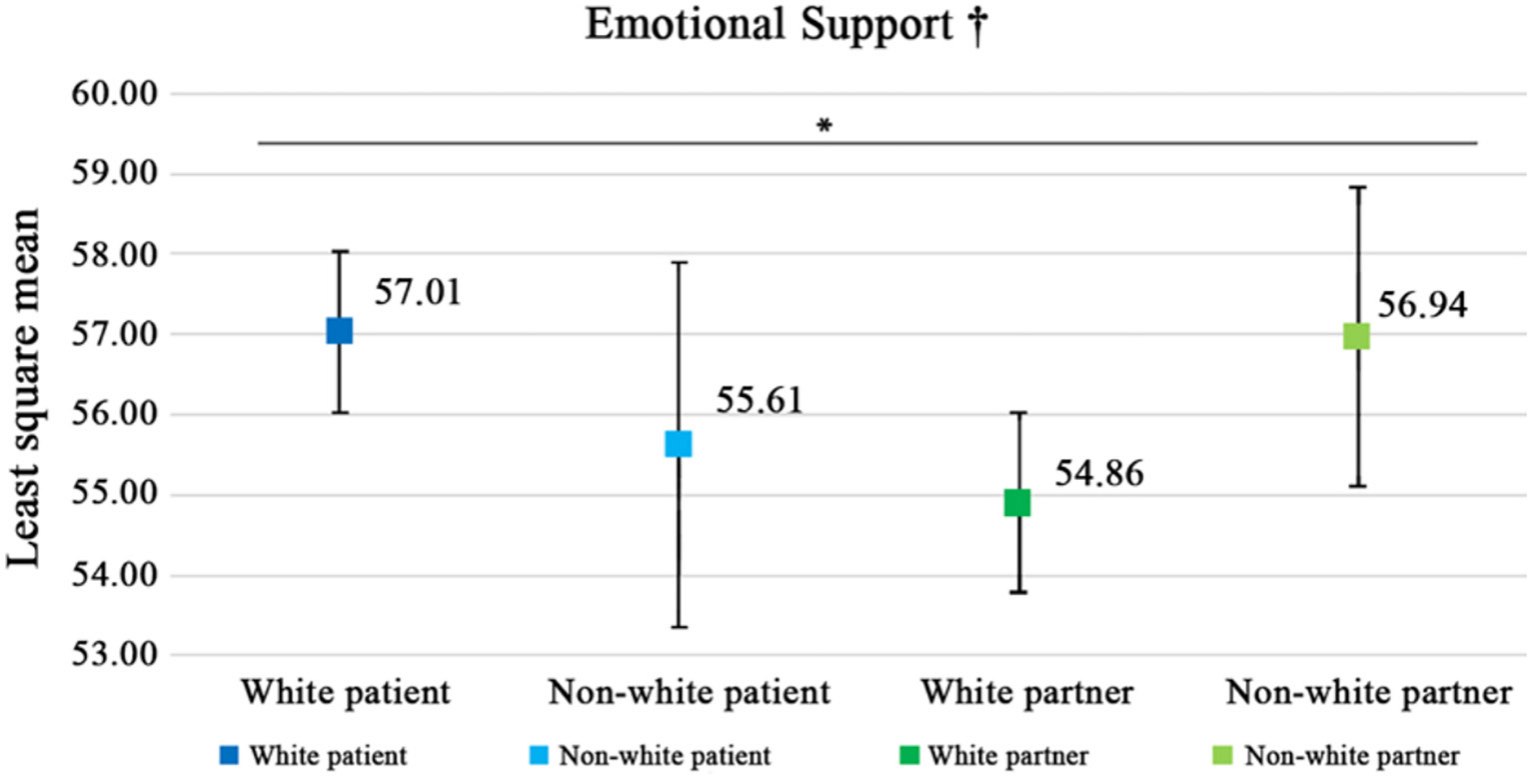
Prostate cancer disparities in emotional support among patients and partners of different races. Results of the least‐square means and 95% confidence interval for MLM after removing the nonsignificant interaction terms: role*race*ADI, role*ADI, and race*ADI. ^†^Higher scores indicated more positive results. *p < 0.05; **p < 0.01; ***p < 0.001.

Results showed significant role‐by‐race interaction effects on emotional support (*p* < 0.05). White patients reported the highest emotional support scores among all groups, and white partners had the lowest emotional support scores (Figure 3).

## 4 DISCUSSION

To our knowledge, this is one of the first studies to comprehensively examine how role, race, and ADI are associated with health outcomes and psychosocial factors among patients with localized PCa and their partners during post‐treatment transition. Expanding the evidence base for PCa disparities beyond the racial differences in survival and incidence and mortality rates, we identified significant disparities not previously researched among patients and their partners by examining the effects of ADI, a neighborhood‐level measure for social determinants of health.

This study made an essential contribution toward understanding the additive effects of race and ADI on health outcomes and psychosocial factors among PCa patients and their partners. Adding to research on racial disparities in PCa incidence and mortality,^40^, ^41^ we found that compared to their white counterparts, non‐white (88.52% Black) patients who recently completed treatment for PCa with curative intent and partners reported better overall, emotional, and functional QOL; more depression; more positive appraisals of illness; and greater self‐efficacy in illness management. The more positive QOL outcomes and psychosocial factors in non‐whites may indicate coping resources that support greater resiliency in non‐whites than their white counterparts during PCa survivorship. However, other researchers have reported that non‐white long‐term PCa survivors have higher incidence and associated mortality than Whites.^42^ Future qualitative research may help to unpack the evidence and understand the findings from this study. Research is also needed to identify the factors that may allow patients and partners to have more positive QOL outcomes, illness appraisals, and confidence in PCa management. Identifying these factors will help develop interventions tailored to the needs of patients and caregivers of different racial groups and support their management of PCa and the effects of treatment for it both short term and long term.

Meanwhile, we found significant ADI effects on social well‐being and PCa‐specific symptoms. ADI measures socioeconomic deprivation at the neighborhood level using 17 indicators, including education, employment, housing quality, and poverty, allowing the identification of PCa disparities that race alone failed to capture.^36^ Compared to those living in less deprived neighborhoods, patients and partners living in more deprived neighborhoods, regardless of race, reported worse social QOL; more bothersome PCa‐specific urinary, hormonal, and sexual symptoms; and less interpersonal support, even after controlling for age, education, ethnicity, employment status (presently working), comorbid conditions (CCI), the type of cancer treatment, and Gleason score. While people in poor neighborhoods reported more financial and neighborhood problems and rated themselves lowest on the ladder of society,^43^ individuals who report that their neighborhoods have more social resources, social order, and social control tend to exchange more social support. Individuals in neighborhoods with more social disorder report providing more support but receiving less.^44^ Findings from our study suggest that health outcomes of patients and partners living in more deprived neighborhoods are impacted both directly by PCa symptoms and indirectly by lack of access to the social support resources that are essential for their well‐being when managing critical health conditions such as PCa. Negative emotional responses and poor QOL are related to poor survival and high cancer mortality,^45^ and poor QOL and symptoms provide useful PCa prognostic information.^46^ It is, thus, important to provide support tailored to the needs of patients and partners living in deprived neighborhoods (e.g., developing new social support network through peer and professional support) to improve their health outcomes and reduce mortality.

Our findings of role effects are consistent with the results from previous research^14^, ^15^; compared to the PCa patients, partners reported better physical QOL, more frequent anxiety and fatigue, more pain, perceived more bother by patients' hormonal symptoms but less bother with patients' urinary and sexual problems, and lower self‐efficacy and instrumental support. Interestingly, white patients reported the highest, whereas white partners reported the lowest emotional support, with non‐white patients and partners reporting emotional support in the middle. Previous research reported that perceived social support reduced caregiver psychological distress,^47^ caregiver engagement with their social networks was positively associated with self‐efficacy in providing care, and caregiver social engagement and self‐efficacy were positively associated with patient health.^48^ Our findings, thus, may suggest that addressing the psychosocial and instrumental support needs of PCa patients' partner caregivers could improve anxiety and somatic symptoms, enhance self‐efficacy, and ultimately improve the health outcomes of patients. Furthermore, nearly all partners in our study were women. Female partners often report an increased prevalence of anxiety disorder and depression and more frequent complaints of somatic discomfort, including fatigue and muscle tension, than male partners.^49^ Future research is needed to pinpoint whether the disparities identified in this study are related to role, gender, or both and identify factors contributing to the disparities. Additional research is also needed to understand the significant differences in patients' and partners' perceived bothers by patients' PCa‐related symptoms.

While further research is needed to understand the mechanisms accounting for the abovementioned PCa disparities, our study calls for targeted multilevel interventions to support patients of different racial backgrounds and their partners living in neighborhoods with different levels of resource deprivation. Current interventions are mostly individual‐ or dyad‐focused; they target improving patients' and partners/caregivers' QOL and symptom management and enhancing their positive appraisals, self‐efficacy, and social support. Nevertheless, tailored multilevel interventions should also integrate strategies of promoting interpersonal support (e.g., support from peers and community members) and enhancing existing resources at the neighborhood level to facilitate symptom management (e.g., access to healthcare and recreation). Integrating social determinants of health data into electronic health records could also help healthcare workers refer patients to neighborhood‐specific resources.

### 4.1 Limitations

This study has the following limitations. First, due to small sample sizes, we categorized patients and partners who self‐reported race as Black, Asian, or “other race” as “non‐white” although 88.52% were Blacks. Next, this study focused on gender‐specific PCa. All patients were male, and nearly all partners were female (except for one partner). Finally, we only included patients who were diagnosed with localized PCa. These factors may limit the generalizability of our findings. Future research with sufficient sample sizes of racial/ethnic minorities and the inclusion of patients with advanced PCa and their caregivers will help examine the granularities of PCa disparities related to role, race, and ADI.

### 4.2 Strengths

This study analyzed the data from a population‐based cohort of patients and partners with more diverse sociodemographic and clinical characteristics compared to those conveniently recruited through cancer centers and hospitals. Typically, recruitment rates are lower in population‐based studies than in clinic‐based studies. Recruitment of patient‐partner dyads into intervention research is challenging, especially during the post‐treatment care transition. However, we have achieved a recruitment rate of 12.78%, similar to rates in other population‐based studies. Our study also used validated measures to assess ADI, health outcomes, and psychosocial factors among patients and partners and controlled for the effects of various demographic and cancer‐related covariates.

## 5 CONCLUSIONS

This study used a multilevel approach to examine the effects of role, race, and ADI on health outcomes and psychosocial factors among patients with PCa and their partners, controlling for various demographic and cancer‐related covariates. Our work contributes to a more comprehensive understanding of PCa disparities than previous research focusing on individual‐ and/or family‐level factors (e.g., race and role). In addition to personal‐ and family‐focused strategies, supportive care programs should be tailored at multiple levels and provide adequate support to address neighborhood resource disparities, and ultimately, to reduce PCa disparities for patients and their partners and improve their health outcomes.

## AUTHOR CONTRIBUTIONS

**Lixin Song:** Conceptualization (lead); data curation (supporting); funding acquisition (lead); investigation (lead); methodology (lead); project administration (lead). **Thomas Keyserling:** Conceptualization (supporting); writing – original draft (equal); writing – review and editing (equal). **Ronald C Chen:** Conceptualization (supporting); methodology (equal); writing – original draft (equal); writing – review and editing (equal). **Chunxuan Ma:** Formal analysis (equal); methodology (equal); software (equal); validation (equal); visualization (equal); writing – original draft (equal); writing – review and editing (equal). **Shenmeng Xu:** Data curation (supporting); project administration (equal); writing – original draft (supporting). **Karl Shieh:** Data curation (supporting); project administration (equal); writing – original draft (supporting). **Gail Fuller:** Investigation (lead); project administration (lead); supervision (lead); writing – review and editing (equal). **Matthew E Nielsen:** Conceptualization (supporting); resources (supporting); writing – original draft (supporting); writing – review and editing (supporting). **Laurel Northouse:** Conceptualization (equal); investigation (supporting); writing – review and editing (equal). **Xianming Tan:** Formal analysis (lead); investigation (supporting); methodology (equal); software (lead); supervision (equal); validation (lead); visualization (equal); writing – original draft (equal); writing – review and editing (equal). **Christine Rini:** Conceptualization (equal); methodology (equal); writing – original draft (equal); writing – review and editing (equal).

## CONFLICT OF INTEREST STATEMENT

The authors declare that they have no conflict of interest.

## ETHICS STATEMENT

Informed consent was obtained from all individual participants involved in the study.

## PRECIS

The health outcomes and stress‐coping related psychosocial factors varied significantly among localized prostate cancer (PCa) patients with different racial backgrounds and their partners living in neighborhoods with different levels of resource deprivation. Integrating neighborhood deprivation measurements allowed the identification of PCa disparities that race alone fails to capture.

## Supporting information


Appendix S1

## Data Availability

The data that support the findings of this study are available from the corresponding author upon reasonable request.
