# Supplementary material for: Role, race, and place: Prostate cancer disparities in Patients' and Partners' health outcomes and psychosocial factors
Source: Cancer Med. 2023 Feb 7;12(8):9857–67. doi: 10.1002/cam4.5646 (PMC10166971; doi:10.1002/cam4.5646)
Supplement: Supplementary file 1 — Appendix S1 [file CAM4-12-9857-s001.docx]

Appendix 1. The Role, Race, and ADI Effects on Prostate Cancer Disparities: The MLM Results

|  |  |  | **White** | **Non-white** | **High ADI** ${}^{1}$  **(More deprived neighborhood)** | **Low ADI**  **(Less deprived neighborhood)** | **MLM Result** ${}^{2}$ | | |
| --- | --- | --- | --- | --- | --- | --- | --- | --- | --- |
|  |  |  |  |  |  |  | **Role** | **Race** | **ADI** |
|  | **Score range** | **Cronbach's alpha** | **(Mean, SD)** | **(Mean, SD)** | **(Mean, SD)** | **(Mean, SD)** | **Estimate** | **Estimate** | **Estimate** |
| **Cancer-related QOL†** |  |  |  |  |  |  |  |  |  |
| FACT-G Total Score |  |  |  |  |  |  |  |  |  |
| Patient | 0~108 | 0.90 | (88.77, 13.92) | (89.67, 13) | (85.83, 15.21) | (91.36, 11.94) | -0.94 | 3.01** | -2.50 |
| Partner |  | 0.91 | (88.56, 14.27) | (90.87, 14.52) | (87.22, 14.98) | (90.48, 13.61) |  |  |  |
| FACT-G Physical Well-being |  |  |  |  |  |  |  |  |  |
| Patient | 0~28 | 0.76 | (23.72, 4.18) | (24.51, 3.8) | (23.08, 4.59) | (24.52, 3.58) | -0.76* | 0.69 | -0.29 |
| Partner |  | 0.79 | (24.42, 3.81) | (24.35, 5.39) | (24.09, 4.38) | (24.63, 4.06) |  |  |  |
| FACT-G Social Well-being |  |  |  |  |  |  |  |  |  |
| Patient | 0~28 | 0.72 | (23.14, 3.75) | (22.69, 4.56) | (22.29, 4.68) | (23.62, 3.17) | 0.02 | 0.15 | -1.05* |
| Partner |  | 0.80 | (22.92, 4.74) | (23.15, 4.09) | (22.39, 4.92) | (23.41, 4.26) |  |  |  |
| FACT-G Emotional Well-being |  |  |  |  |  |  |  |  |  |
| Patient | 0~24 | 0.83 | (20.37, 3.93) | (20.93, 4.02) | (19.77, 4.41) | (21.05, 3.48) | 0.39 | 1.07** | -0.48 |
| Partner |  | 0.77 | (19.62, 3.73) | (20.44, 4.38) | (19.52, 4.24) | (20.01, 3.6) |  |  |  |
| FACT-G Functional Well-being |  |  |  |  |  |  |  |  |  |
| Patient | 0~28 | 0.82 | (21.54, 5.14) | (21.53, 5.13) | (20.69, 5.52) | (22.18, 4.72) | -0.56 | 1.13* | -0.64 |
| Partner |  | 0.83 | (21.53, 4.88) | (22.93, 4.71) | (21.21, 5.01) | (22.32, 4.69) |  |  |  |
| **General Symptoms (PROMIS)‡** |  |  |  |  |  |  |  |  |  |
| Anxiety |  |  |  |  |  |  |  |  |  |
| Patient | 36.3~82.7 | 0.93 | (45.63, 8.84) | (45.6, 8.95) | (47.31, 9.97) | (44.34, 7.68) | -2.94*** | -1.66 | 0.82 |
| Partner |  | 0.93 | (49.62, 9.65) | (47.88, 9.73) | (49.74, 9.04) | (48.9, 10.12) |  |  |  |
| Depression |  |  |  |  |  |  |  |  |  |
| Patient | 37.1~81.1 | 0.94 | (44.98, 8.49) | (44.59, 8.91) | (46.05, 9.43) | (44.01, 7.77) | -0.85 | -1.95* | 0.36 |
| Partner |  | 0.94 | (46.77, 8.85) | (44.51, 8.93) | (46.54, 9.26) | (46.07, 8.59) |  |  |  |
| Pain |  |  |  |  |  |  |  |  |  |
| Patient | 41~78.3 | 0.94 | (48.35, 8.72) | (48.32, 9.34) | (49.76, 9.53) | (47.27, 8.15) | -1.53* | -0.59 | 0.82 |
| Partner |  | 0.96 | (50.12, 9.43) | (50.24, 10.8) | (51.33, 10.03) | (49.22, 9.42) |  |  |  |
| Sleep |  |  |  |  |  |  |  |  |  |
| Patient | 28.9~76.5 | 0.93 | (49.35, 9.49) | (49.6, 9.84) | (50.75, 9.83) | (48.39, 9.24) | 0.88 | -1.67 | 0.14 |
| Partner |  | 0.93 | (49.82, 9.41) | (47.5, 11.03) | (49.33, 9.63) | (49.3, 9.96) |  |  |  |
| Fatigue |  |  |  |  |  |  |  |  |  |
| Patient | 29.4~83.2 | 0.85 | (46.9, 9.31) | (46.23, 9.73) | (48.39, 10.06) | (45.5, 8.68) | -1.77* | -0.27 | 0.15 |
| Partner |  | 0.87 | (48.54, 9.15) | (49.73, 11.31) | (49.26, 9.62) | (48.47, 9.67) |  |  |  |
| **PCa Specific Symptoms: EPIC†**${}^{3}$ |  |  |  |  |  |  |  |  |  |
| Urinary |  |  |  |  |  |  |  |  |  |
| Patient | 0~100 | 0.78 | (67.43, 19.26) | (70.04, 21.94) | (65.47, 20.53) | (69.89, 19.17) | -10.53*** | 0.72 | -4.91* |
| Partner |  | NA | (78.47, 27.02) | (77.42, 32.55) | (75.64, 30.04) | (79.84, 26.89) |  |  |  |
| Bowel |  |  |  |  |  |  |  |  |  |
| Patient | 0~100 | 0.79 | (90.63, 14.77) | (92.42, 11.27) | (88.39, 16.27) | (93.04, 11.78) | -1.40 | 0.89 | -3.06 |
| Partner |  | NA | (92.34, 17.88) | (91.13, 21.28) | (89.19, 21.80) | (94.35, 15.49) |  |  |  |
| Sexual |  |  |  |  |  |  |  |  |  |
| Patient | 0~100 | 0.87 | (22.69, 25.45) | (21.31, 23.48) | (18.25, 21.05) | (25.52, 27.27) | -43.20*** | -2.94 | -6.09* |
| Partner |  | NA | (66.30, 37.40) | (60.89, 42.61) | (62.72, 39.63) | (66.40, 38.18) |  |  |  |
| Hormonal |  |  |  |  |  |  |  |  |  |
| Patient | 0~100 | 0.58 | (87.33, 13.86) | (85.08, 17.45) | (83.35, 15.25) | (89.5, 13.79) | 8.37*** | -0.76 | -4.55* |
| Partner |  | NA | (77.27, 28.44) | (76.21, 32.15) | (72.03, 30.87) | (80.81, 27.45) |  |  |  |
| **Appraisals**† |  |  |  |  |  |  |  |  |  |
| Overall Appraisal of Illness Score${}^{4}$ |  |  |  |  |  |  |  |  |  |
| Patient | 1~5 | 0.85 | (3.75, 0.66) | (3.87, 0.67) | (3.67, 0.68) | (3.86, 0.63) | -0.06 | 0.20** | -0.11 |
| Partner |  | 0.86 | (3.77, 0.62) | (3.93, 0.7) | (3.74, 0.63) | (3.87, 0.64) |  |  |  |
| **Coping Resources**† |  |  |  |  |  |  |  |  |  |
| Cancer Self-Efficacy Scale Score |  |  |  |  |  |  |  |  |  |
| Patient | 9~90 | 0.93 | (80.36, 9.51) | (83, 9.22) | (79.8, 10.39) | (81.83, 8.68) | 1.84* | 2.74* | -0.84 |
| Partner |  | 0.95 | (78.33, 11.45) | (80.08, 12.3) | (78.55, 10.9) | (78.83, 12.15) |  |  |  |
| **Social Support†** |  |  |  |  |  |  |  |  |  |
| Informational Support |  |  |  |  |  |  |  |  |  |
| Patient | 23.7~69.1 | 0.91 | (56.88, 7.68) | (57.83, 9.7) | (56.88, 8.7) | (57.26, 7.75) | 0.39 | 1.49 | -0.50 |
| Partner |  | 0.94 | (56.38, 8.83) | (57.62, 9.06) | (56.31, 8.93) | (56.99, 8.86) |  |  |  |
| Instrumental Support |  |  |  |  |  |  |  |  |  |
| Patient | 27~65.6 | 0.90 | (60.66, 5.68) | (60.2, 6.6) | (60.6, 5.91) | (60.52, 5.89) | 4.48*** | 0.38 | -0.11 |
| Partner |  | 0.95 | (55.65, 8.45) | (56.5, 7.83) | (55.54, 8.57) | (56.1, 8.11) |  |  |  |
| Interpersonal Support |  |  |  |  |  |  |  |  |  |
| Patient | 4~16 | 0.79 | (14.47, 2.02) | (14.11, 2.85) | (13.96, 2.56) | (14.72, 1.9) | 0.11 | -0.09 | -0.44* |
| Partner |  | 0.84 | (14.26, 2.22) | (14.21, 2.32) | (14.08, 2.39) | (14.41, 2.1) |  |  |  |

Footnote:

1. Result of the final parsimonious models after removing nonsignificant 2-way and 3-way interactions.
2. Referents – Role effect: partner; Race effect: white; ADI effect: low ADI.
3. ADI (area deprivation index) was categorized into two groups, high ADI (ADI > 3, more deprived neighborhoods) and low ADI (ADI <= 3, less deprived).
4. †: Higher scores indicated more positive results; ‡: Higher scores indicated more negative results.
5. EPIC scores for partners ranged from 1 to 5. We standardized the partners’ scores using the same approach as recommended by EPIC 26 to be consistent with patients’ scores (range, 0-100).
6. *: *p*<.05, **: *p*<.01, ***: *p*<.001. *p*< .05
7. Taking PROMIS anxiety symptom as an interpretation example: The MLM analysis showed significant role effect (*p* <.001), i.e., partners were 3.00 points higher in their anxiety scores as compared to patients. But the MLM analysis did not find significant race effect (*p* =.10) and ADI effect (*p* =.24), indicating that the anxiety scores were similar among white vs. nonwhite dyads and among dyads in high ADI vs. low ADI.

Appendix 2. Prostate Cancer Disparities in Emotional Social Support among Patients and Their Partners: The MLM Results

|  |  |  |  |  |  |  | **MLM Result** ${}^{2}$ | | | | | | | | | | |
| --- | --- | --- | --- | --- | --- | --- | --- | --- | --- | --- | --- | --- | --- | --- | --- | --- | --- |
| **Outcomes** |  |  | **White** | **Non-white** | **High ADI** ${}^{1}$ **(N=118)** | **Low ADI**  **(N=155)** | **Role** | | **Race** | | | **ADI** | | | **Role*Race** | | |
|  |  |  |  |  |  |  |  |  |  |  |  |  |  |  |  |  |  |
|  | **Score range** | **Cronbach's alpha** | **Mean (SD)** | **Mean (SD)** | **Mean (SD)** | **Mean (SD)** | **Estimate** | **P-value** | | **Estimate** | **P-value** | | **Estimate** | **P-value** | **Estimate** | | **P-value** |
| **Coping resources - Social support: Emotional Support†** | | | |  |  |  |  |  | |  |  | |  |  |  |  |  |
|  |  |  |  |  |  |  |  |  | |  |  | |  |  |  |  |  |
| Patient | 24.7~63.5 | 0.95 | 57.11(7.06) | 55.55(8.89) | 56.63(8.56) | 56.86(6.64) | 0.41 | 0.62 | | 0.35 | 0.69 | | 0.77 | 0.97 | White patient | 57.01 | <0.05 |
|  |  |  |  |  |  |  |  |  |  |  |  |  |  |  | Non-white patient | 55.61 |  |
| Partner |  | 0.95 | 54.87(7.97) | 56.51(7.80) | 55.30(8.21) | 55.31(7.77) |  |  |  |  |  |  |  |  | White partner | 54.86 |  |
|  |  |  |  |  |  |  |  |  |  |  |  |  |  |  | Non-white partner | 56.94 |  |

Footnote:

1. Result of the final parsimonious models after removing nonsignificant interaction terms: role * race *ADI, role*ADI, and race*ADI.
2. Referents – Role effect: partner; Race effect: white; ADI effect: low ADI.
3. †: Higher scores indicated more positive results.
